# Genome-wide expression analysis of salt-stressed diploid and autotetraploid *Paulownia tomentosa*

**DOI:** 10.1371/journal.pone.0185455

**Published:** 2017-10-19

**Authors:** Zhenli Zhao, Yongsheng Li, Haifang Liu, Xiaoqiao Zhai, Minjie Deng, Yanpeng Dong, Guoqiang Fan

**Affiliations:** 1 Institute of Paulownia, Henan Agricultural University, Zhengzhou, Henan, China; 2 Forestry Academy of Henan, Zhengzhou, Henan, P.R. China; Key Laboratory of Horticultural Plant Biology (MOE), CHINA

## Abstract

*Paulownia tomentosa* is a fast-growing tree species with multiple uses. It is grown worldwide, but is native to China, where it is widely cultivated in saline regions. We previously confirmed that autotetraploid *P*. *tomentosa* plants are more stress-tolerant than the diploid plants. However, the molecular mechanism underlying *P*. *tomentosa* salinity tolerance has not been fully characterized. Using the complete *Paulownia fortunei* genome as a reference, we applied next-generation RNA-sequencing technology to analyze the effects of salt stress on diploid and autotetraploid *P*. *tomentosa* plants. We generated 175 million clean reads and identified 15,873 differentially expressed genes (DEGs) from four *P*. *tomentosa* libraries (two diploid and two autotetraploid). Functional annotations of the differentially expressed genes using the Gene Ontology and Kyoto Encyclopedia of Genes and Genomes databases revealed that plant hormone signal transduction and photosynthetic activities are vital for plant responses to high-salt conditions. We also identified several transcription factors, including members of the AP2/EREBP, bHLH, MYB, and NAC families. Quantitative real-time PCR analysis validated the expression patterns of eight differentially expressed genes. Our findings and the generated transcriptome data may help to accelerate the genetic improvement of cultivated *P*. *tomentosa* and other plant species for enhanced growth in saline soils.

## Introduction

Plants, as sessile organisms, must cope with a multitude of biotic and abiotic stresses throughout their life cycle. Among the abiotic stresses (e.g., high salinity, extreme temperatures, drought, heavy metal contamination, and nutrient deficiency), salt stress is the most serious threat to sustainable agricultural productivity worldwide, but especially in developing countries [1]. High salinity affects more than 800 million ha of land, which represents more than 6% of the total global land area. This area includes 45 million ha (i.e., approximately 20%) of the 230 million ha of irrigated land [2]. Soil salinity affects plants in two ways: One, high salt concentrations make it difficult for roots to take up enough water; and two, high intracellular salt concentrations can be toxic to plants.

*Paulownia tomentosa* is indigenous to China, where it grows in the plains and at altitudes up to 2000 m [3]. This tree species has been introduced to Japan and Southeast Asia, Australia, Brazil, Europe, and North and Central America. *P*. *tomentosa* is an economically important tree species in the family Scrophulariaceae. It is used to make aircraft parts, toys, musical instruments, plywood, furniture, and medicinal compounds [4]. Paulownia species are also useful as fertilizers and fodder [3]. Its benefits to the environment are partly based on the fact it can grow in nutrient-poor soil and has a deep root system [5, 6]. These characteristics make *P*. *tomentosa* potentially useful for reforestation of areas with nutrient-poor soils [7]. *P*. *tomentosa* trees can adapt to different soil conditions and climates [8, 9], and are planted mainly in salinized soil, saline-alkali soil [10], or in regions with limited irrigation water [11]. This suggests that this tree species may be genetically adapted to saline conditions and other stresses. Thus, the molecular mechanisms regulating the adaptation of *P*. *tomentosa* to salt stress should be characterized.

An autotetraploid *P*. *tomentosa* line has been generated from a diploid line using colchicines [12]. Compared with diploid *P*. *tomentosa* plants, the autotetraploid plants exhibit higher net photosynthetic rates and better wood physical properties [13, 14]. We previously analyzed the morphology and physiology of *P*. *tomentosa* trees, and confirmed that autotetraploid *P*. *tomentosa* trees are more stress resistant than the diploid trees [15–17].

Before the *Paulownia fortunei* genome was sequenced, several studies of the *P*. *tomentosa* transcriptome [9, 18–20], miRNAs [21–23], and proteome were conducted [24]. These studies were focused mainly on diploid and autotetraploid *P*. *tomentosa*, *Paulownia* witches’ broom disease, and drought, and salt stress against the background of the transcriptome data. The genomes of many plant species have now been sequenced, including *Phoenix dactylifera*L. [25], *Malus × domestica*Borkh [26], and *Populustrichocarpa* [27], and the increasing availability of sequenced plant genomes has encouraged researchers to analyze non-model plants using high-throughput sequencing techniques. Sequenced genomes also improve the accuracy of gene annotations. However, until now, there have been no published investigations focused on *Paulownia* species, including *P*. *tomentosa*, based on the *P*. *fortunei* genome. Additionally, there has been very little research into the effects of salt stress on *P*. *tomentosa* [20], and the mechanism regulating salt tolerance has not been elucidated.

In this study, we used an RNA-sequencing (RNA-Seq) technique based on the *P*. *fortunei* genome to analyze the transcript profiles of salt-stressed and control diploid and autotetraploid *P*. *tomentosa* lines. The diploid and autotetraploid *P*. *Tomentosa* trees were acquired from the same tissue culture, and may represent biological replicates. The autotetraploid *P*. *tomentosa* line was considered to be more appropriate for investigating plant responses to salt stress than the natural diploid line. The Gene Ontology (GO) and Kyoto Encyclopedia of Genes and Genomes (KEGG) databases were used to analyze the differentially expressed genes (DEGs) among four pairwise comparisons. The expression patterns of eight DEGs were confirmed with a quantitative real-time polymerase chain reaction (qRT-PCR) assay. Additionally, bioinformatics analyses helped to elucidate plant hormone signal transduction pathways (abscisic acid and cytokinin) and photosynthetic activities. Furthermore, some transcription factors (TFs), such as bHLH, MYB, and NAC, were observed to be associated with salt stress responses or ploidy levels. To the best of our knowledge, this is the first report comparing the diploid and autotetraploid *P*. *tomentosa* lines based on the whole genome sequence of *P*. *fortunei*. The genetic basis of *P*. *tomentosa* salt tolerance was also investigated.

## Materials and methods

### Plant materials and salt treatments

All plant materials used in this study were obtained from the Institute of Paulownia, Henan Agricultural University, Zhengzhou, Henan Province, China. The tissue-cultured diploid and autotetraploid *P*. *tomentosa* seedlings were grown for 30 days at 25 ± 2°C on half-strength Murashige and Skoogmedium [28] with a 16-h photoperiod (light intensity: 130 μmol m^−2^ s^−1^). The test-tube plantlets were then transferred outdoors into nutritive bowls containing normal garden soil supplemented with 0.11% (quality score) NaCl solution, and grown for another 30 days. Consistently growing healthy seedlings were transferred to larger nutritive bowls with trays underneath. After 50 days, the uniformly growing seedlings were irrigated with 0% (control) or 0.4% (quality score) NaCl solutions. For the salt treatment, NaCl was weighed and divided into three equal parts, which were dissolved in water and poured into the bowls. The water that accumulated in the trays below the bowls was poured back into the bowls. The seedlings were treated with the control or salt solution every 3 days. After all of the salt water was added back into the bowls, the plants were watered every 2 days to maintain the soil moisture content at 75%. After 15 days, the second pair of leaves (fully expanded leaves) from the apex shoot of the control and salt-treated diploid and autotetraploid *P*. *tomentosa* plants were collected and immediately frozen in liquid nitrogen. The samples were stored at −80 ± 2°C. The control diploid and autotetraploid *P*. *tomentosa* samples were named PT2 and PT4, respectively, while the salt-treated diploid and autotetraploid samples were named PT2S and PT4S, respectively.

### RNA extraction, cDNA library preparation, and sequencing

Total RNA was extracted from PT2, PT4, PT2S, and PT4S leaf samples using the Plant RNA Isolation Kit (AutoLab, Beijing, China) and concentrated using an RNeasy MinElute Cleanup Kit (Qiagen, Valencia, CA, USA). The RNA was then treated with DNase I to eliminate any contaminating genomic DNA. Oligo-d(T) magnetic beads were used to isolate mRNA from the purified total RNA. The mRNA was fragmented in fragmentation buffer (Life Technologies, Beijing, China), and then used as the template for cDNA synthesis. Short fragments were purified and resolved with ethidium bromide buffer for the subsequent end-repair and A (adenine)-tailing. The short fragments were linked to adapters, and analyzed by agarose gel electrophoresis. The suitable fragments were selected as templates for qRT-PCR. The quality and quantity of the sample libraries were analyzed using an Agilent 2100 Bioanalyzer (Agilent Technologies, Palo Alto, CA, USA). The qRT-PCRs were conducted using an ABI StepOnePlus Real-Time PCR System (ABI, New York, NY, USA). Finally, the libraries were sequenced on an IlluminaHiSeq^™^ 2000 platform.

### Quality control and alignment/mapping of clean reads

The original image data were transformed into sequence data via base calling. These raw reads were filtered using the SOAPnuke program, as previously described [20], and the remaining “clean” reads were used in the downstream bioinformatics analyses. The quality of the clean reads data was assessed using base composition and quality distribution charts. The remaining reads were aligned to the *P*. *fortunei* genome and gene sequences using BWA and Bowtie, respectively. The statistics of alignment results will be presented for each reference. During the RNA-sequencing experiment, transcripts were chemically fragmented and then sequenced. The distribution of reads on the genes was used to evaluate the randomness of the RNA fragmentation associated with the constructed libraries [29].

### Gene expression analysis

Gene expression levels were determined with the RSEM (RNA-Seq by Expectation Maximization) software package. The FPKM method (fragments per kb per million fragments) was used to calculate expression levels and to identify DEGs [30]. In multiple tests, the false discovery rate (FDR) was applied to determine the threshold *p*-value [31]. FDR ≤ 0.001 and absolute log_2_ ratio ≥ 1 were used as the threshold to evaluate the significance of gene expression differences.

Correlations between two samples were assessed based on the FPKM result. Ideally, the squared correlation value should be ≥ 0.92 according to the standard recommended by the ENCODE. The distances between expressed genes were calculated according to the Euclidean method. The sum of squared deviations algorithm was used to calculate the distance between samples so that a cluster tree could be built. The DEGs between two libraries were identified using the Audic-Claverie statistic [32]. The DEGs were functionally annotated based on GO terms and KEGG pathways.

### Analysis of differentially expressed genes

The DEGs were first mapped to GO terms in the GO database, and the number of genes for each term was calculated. Then, the hypergeometric test was used to identify significantly enriched GO terms (http://www.geneontology.org/). The calculated *p-*values were adjusted using the Bonferroni correction [33], with a corrected *p-*value ≤ 0.05 used as the threshold. GO terms that fulfilled, this condition were defined as significantly enriched, and were considered to describe the main biological functions of the DEGs. The WEGO program [34] was used to functionally classify the DEGs and to assess the distribution of gene functions in *P*. *tomentosa* at a macro level. The KEGG database was used to identify enriched pathways associated with the DEGs [33].

TFs generally contain a DNA-binding domain and a *trans*-acting functional domain [35]. We used the hmmsearch program to search the HMMS database for domain characteristics, and to predict if a gene encoded a TF. Additionally, the TopHat program was used [36] to analyze the various types of alternative splicing (AS)-related clean reads mapped to the *P*. *fortunei* reference genome in the four samples. The AS events that were identified in both replicates were considered as stable events.

### Comparison of gene expression profiles among different samples

**Fig 1:**
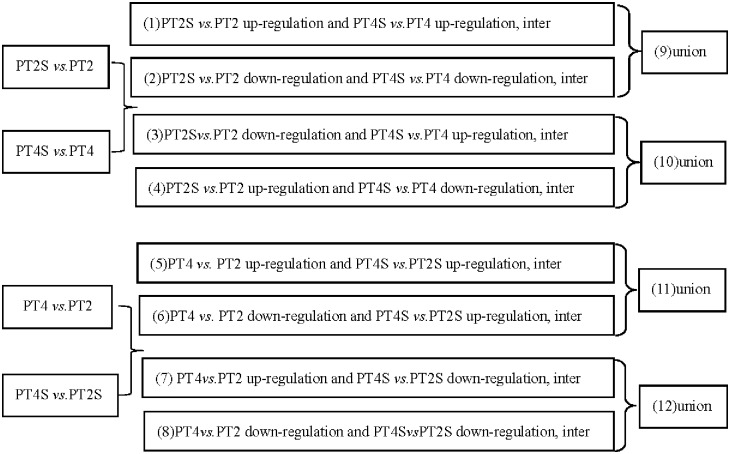
Comparison schemes of the four samples.

The following pairwise comparisons of DEGs were evaluated (Fig 1): Comparison A: co-up- and co-down-regulation in PT2S *vs*. PT2 and PT4S *vs*. PT4; Comparison B: down-regulation in PT2S *vs*. PT2, and up-regulation in PT4S *vs*. PT4 as well as up-regulation in PT2S *vs*. PT2, and down-regulation in PT4S *vs*. PT4; Comparison C: up-regulation in PT4 *vs*. PT2 and PT4S *vs*. PT2S as well as down-regulation in PT4 *vs*. PT2 and up-regulation in PT4S *vs*. PT2S; and Comparison D: up-regulation in PT4 *vs*. PT2, and down-regulation in PT4S *vs*. PT2S as well as down-regulation in PT4 *vs*. PT2 and PT4S *vs*. PT2S.

PT2 and PT4 represent the diploid and autotetraploid *P*. *tomentosa* with salt-untreated, PT2S and PT4S represents the diploid and autotetraploid *P*. *tomentosa* with salt-treated (0.4% Nacl).(1) Differentially expressed genes (DEGs) were co-up-regulation between the PT2S *vs*. PT2 and PT4S *vs*. PT4 comparison to screen for gene from both diploid and autotetraploid *P*. *tomentosa* after salt treatment were strengthened. (2) DEGs co-down-regulation in the PT2S *vs*. PT2 and PT4S *vs*. PT4 comparison. (3) DEGs down-regulation in the PT2S *vs*. PT2 comparison, but up-regulation in the PT4S *vs*. PT4 comparison. (4) DEGs up-regulation in the PT2S *vs*. PT2 comparison, but down-regulation in the PT4S *vs*. PT4 comparison. (5) DEGs were co-up-regulation between the PT4 *vs*. PT2 and PT4S *vs*. PT2S comparison. (6) DEGs down-regulation in the PT4 *vs*. PT2 comparison but up-regulation in the PT4S *vs*. PT2S comparison. (7) DEGs up-regulation in the PT4 *vs*. PT2 comparison but down-regulation in the PT4S *vs*. PT2S comparison. (8)DEGs co-down-regulation in the PT4 *vs*. PT2 and PT4S vs. PT2S comparison. Comparison A: (1) and (2). Comparison B: (3) and (4). Comparison C: (5) and (6). Comparison: from (7) and (8).

### Quantitative real-time polymerase chain reaction analysis of differentially expressed genes

Total RNA that was extracted from the PT2 and PT4 leaf samples as described above. Three independent biological samples of each were employed in this analysis. The SsoAdvanced^™^ SYBR^®^ Green Supermix (Bio-Rad, Hercules, CA, US) was used for the qRT-PCRs. The amplification reactions were performed as follows: 95°C for 3 min, followed by 40 cycles of 95°C for 10 s and 55°C for 30 s, and derived melting curves were obtained. The reactions were performed on a CFX96^™^ Real-Time System (Boi-Rad, Hercules, CA, USA) and all reactions were run in triplicate. 18S rRNA was used as an internal reference gene. The relative expression levels were calculated using the 2^-ΔΔCt^ method. The primers used for the qPCR reactions were listed in Table 1.

**Table 1:** Primers of qRT-PCR for validation of the selected genes.

Gene ID	Description	Primers (5’-3’)
PAU016338.1	predicted protein	F: GCTAACAAGGAACTGAAT: AATTGAACTGTGTATGCT
PAU023935.1	phosphoinositide phospholipase C 2 isoform 1	F: GCCACAATTCTTATCTGA: CAATAACTCTTACACCTCTA
PAU024013.1	zinc finger A20 and AN1 domain-containing	F: CTATGAAGCAAGAACAAG: ATAACGATAGCAACATCT
PAU022560.1	fructose 1,6 bisphosphate aldolase class 1	F: GATATTATTGGATGGTGAACA: CTCAGCAAGGTAGAAGAA
PAU015218.1	probable serine/threonine-protein kinase Cx32	F: TTGCTGTTAAGAAGTTGAA: CACCAGATTAGGATGAGA
PAU018076.1	hypothetical protein PRUPE_ppa005569mg	F: TTGAGGATGGAAGTGATA: AATACTGACCTTATGCTTAG
PAU000558.1	putative receptor-like serine-threonine protein kinase	F: GCTGCTTCTTATTCTTAT: AATGATTCCAACTATTCC
PAU011992.1	hypothetical protein PRUPE_ppa005598mg	F: AGCCAAGAAGGTTATTATTAC: GAGAGTAGTCCTGTTCATT

## Results

### Sequencing of mRNA and alignments with the reference genes and genome

**Fig 2:**
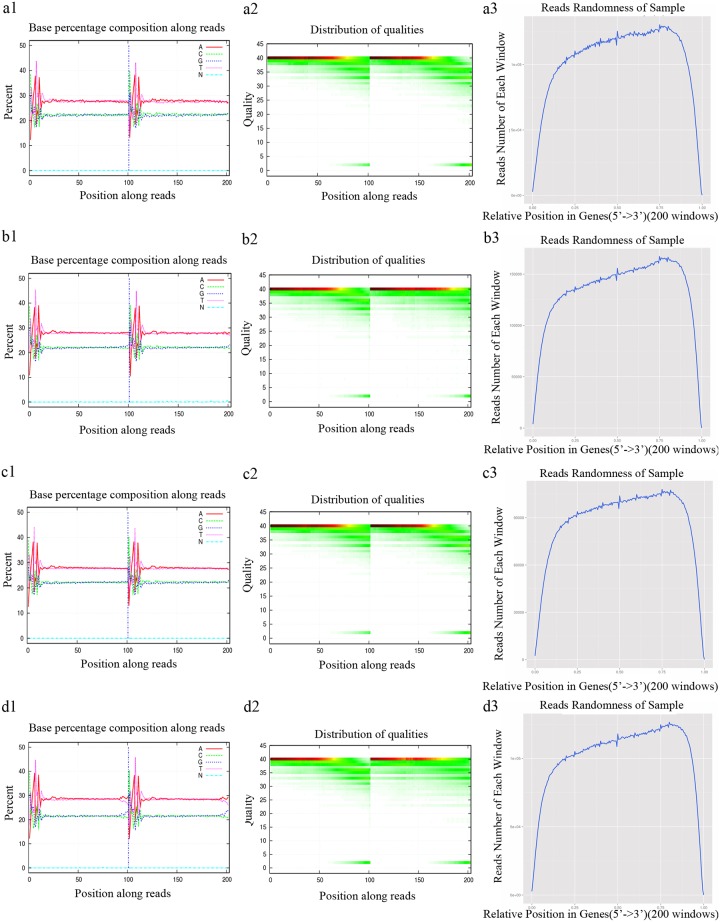
Sequencing data assessment. Base composition of clean data in PT2 (a1), PT2S (b1), PT (c1), and PT4S (d1); base quality of clean data in PT2 (a2), PT2S (b2), PT4 (c2), and PT4S (d2); reads distribution on PT2 (a3), PT2S (b3), PT4 (c3), and PT4S (d3) genes.

Approximately 187 million raw reads were generated for the four sequenced libraries (PT2, PT2S, PT4, and PT4S). Low-quality reads as well as reads with adapter sequences or several unknown bases were eliminated. The quality of the remaining clean reads was assessed using base composition and quality distribution charts. We observed a balanced base composition, with a T curve that was in accordance with the A curve, as well as a satisfactory base composition, high quality sequences, and base ratios that were mostly > 20 (Fig 2). The clean reads were evenly distributed on the genes as indicated by the assessment of the randomness. A total of 175 million clean reads (43,592,404 from PT2, 52,337,180 from PT2S, 33,909,164 from PT4, and 45,725,468 from PT4S) were generated with Q20 of 98.2% (PT2), 96.86% (PT2S), 98.17% (PT4), and 97.24% (PT4S), and Q30 of 95.1% (PT2), 93.12% (PT2S), 95.16% (PT4), and 93.17% (PT4S). The GC content was 44.7% (PT2), 45.15% (PT2S), 44.68% (PT4), and 43.29% (PT4S). These results suggested that high-quality sequencing data were obtained. The clean reads were mapped to the *P*. *fortunei* genes and genome with mapping means of 48.92% and 71.63% respectively (Table 2). We observed that 8.95–17.62% of the total mapped clean reads were aligned to two or more positions, and were considered multi-position matches. Multi-position matches can be problematic during transcriptome-level studies, because they are affected by read complexity and length, Therefore, we excluded the multi-position reads from subsequent analyses to decrease the error rate. The unmapped reads may represent novel genes.

**Table 2:** Statistics of alignment (map to reference gene and genome) from the four libraries (exposed to salt stress for 15 days).

Mapping Type	PT2		PT2S		PT4		PT4S	
	map to reference gene	map to reference genome	map to reference gene	map to reference genome	map to reference gene	map to reference genome	map to reference gene	map to reference genome
**Total clean Reads**	43592404	43592404	52337180	52337180	33909164	33909164	45725468	45725468
**Total BasePairs**	4402832804	4402832804	5286055180	5286055180	3424825564	3424825564	4618272268	4618272268
**Total Mapped Reads**	20876540 (47.89%)	33862719 (77.68%)	26809022 (51.22%)	36218429 (69.20%)	17561062 (51.79%)	25551420 (75.35%)	20476884 (44.78%)	29388276 (64.27%)
**Perfect Match**	11357231 (26.05%)	18314073 (42.01%)	13019282 (24.88%)	17207067 (32.88%)	9174493 (27.06%)	13318604 (39.28%)	9426154 (20.61%)	12806562 (28.01%)
**Mismatch**	9519309 (21.84%)	15548646 (35.67%)	13789740 (26.35%)	19011362 (36.32%)	8386569 (24.73%)	12232816 (36.08%)	11050730 (24.17%)	16581714 (36.26%)
**Unique Match**	13702658 (31.43%)	28050402 (64.35%)	17735298 (33.89%)	31131225 (59.48%)	11585642 (34.17%)	22045482 (65.01%)	13482456 (29.49%)	25295986 (55.32%)
**Multi-position Match**	7173882 (16.46%)	5812317 (13.33%)	9073724 (17.34%)	5087204 (9.72%)	5975420 (17.62%)	3505938 (10.34%)	6994428 (15.30%)	4092290 (8.95%)
**Total Unmapped Reads**	22715862 (52.11%)	9729685 (22.32%)	25528156 (48.78%)	16118751 (30.80%)	16348100 (48.21%)	8357744 (24.65%)	25248582 (55.22%)	16337192 (35.73%)

### Overall analysis of gene expression

**Fig 3:**
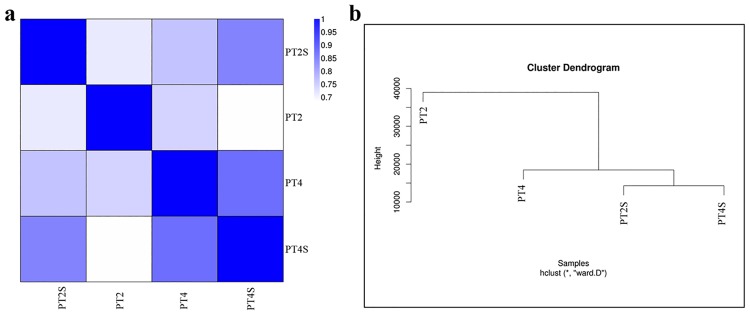
Heatmap of all correlation values (a) and cluster tree of the four accessions (PT2, PT2S, PT4, and PT4S) (b).

**Fig 4:**
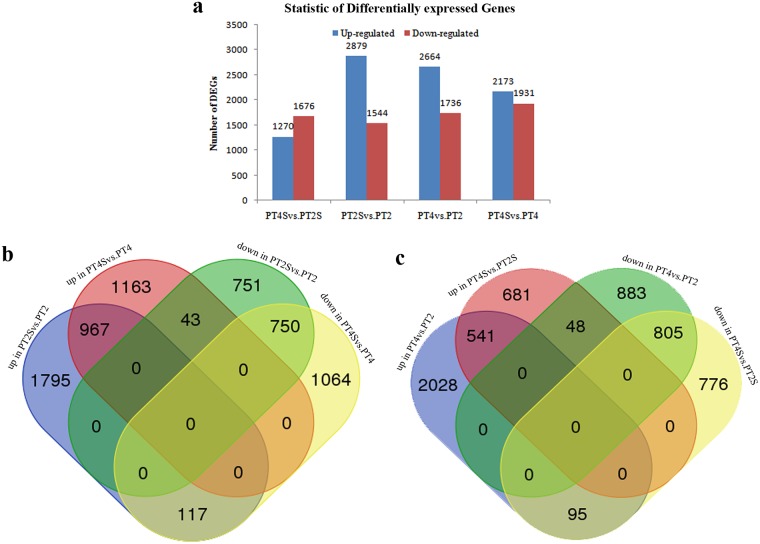
(a) The differently expressed genes in PT4S vs. PT2S, PT2S vs. PT2, PT4 vs. PT2, and PT4S vs. PT4. (b, c) Details of the comparison schemes, up: up-regulated, down: down-regulated.

The correlation value for PT4 and PT4S was > 0.86, indicating the molecular factors responsive to salt stress partially overlapped. The correlations among the other samples are indicated in Fig 3A and S1 Table. The correlation values were close to 1 for the same samples in almost every experiment, indicating that our sequencing data were reliable and the selected samples were reasonable. The clustering of the expression profiles for the salt stress and control treatments of the four samples is presented in Fig 3B. The dendrogram revealed that PT2S and PT4S sequences tended to cluster together. We detected 4,423 DEGs between PT2S and PT2, and 4,104 DEGs between PT4S and PT4. Additionally, 2,946 and 4,400 DEGs were observed for the comparisons between PT4S and PT2S and between PT4 and PT2, respectively (Fig 4A, S2 Table).

In the pairwise comparisons of specific sets of DEGs, we detected 1,717 DEGs for Comparison A, which may be related to *P*. *tomentosa* responses to saline conditions (Fig 4B, S3 Table); and 160 DEGs for Comparison B, which may be relevant to the differences in the salt-induced responses of PT2 and PT4. Furthermore, Comparisons C and D revealed 589 (17.50%) and 900 (26.74%) DEGs, respectively (Fig 4C, S3 Table), which may help to explain why PT4 is more salt-tolerant than PT2.

### Analysis of differentially expressed genes using the GO and KEGG databases

The DEGs detected in Comparisons A, B, C, and D (Fig 1) were categorized into the three main GO categories using the Blast2GO program. Comparisons A, B, C, and D comprised 1,117, 116, 395, and 614 DEGs classified into 40, 27, 36, and 36 functional groups, accounting for 65.02%, 72.50%, 66.95%, and 68.15% of the total DEGs, respectively (S1 Fig, S3 and S4 Tables). Under the biological process category, metabolic process consisted of 561, 64, 179, and 283 DEGs, and cellular process included 467, 50, 146, and 235 DEGs for Comparisons A, B, C, and D, respectively. Under the cellular component category, cell and cell part each consisted of 504, 47, 204, and 299 DEGs for Comparisons A, B, C, and D, respectively. Under the molecular function category,catalytic activity and binding contained the highest numbers of DEGs.

**Fig 5:**
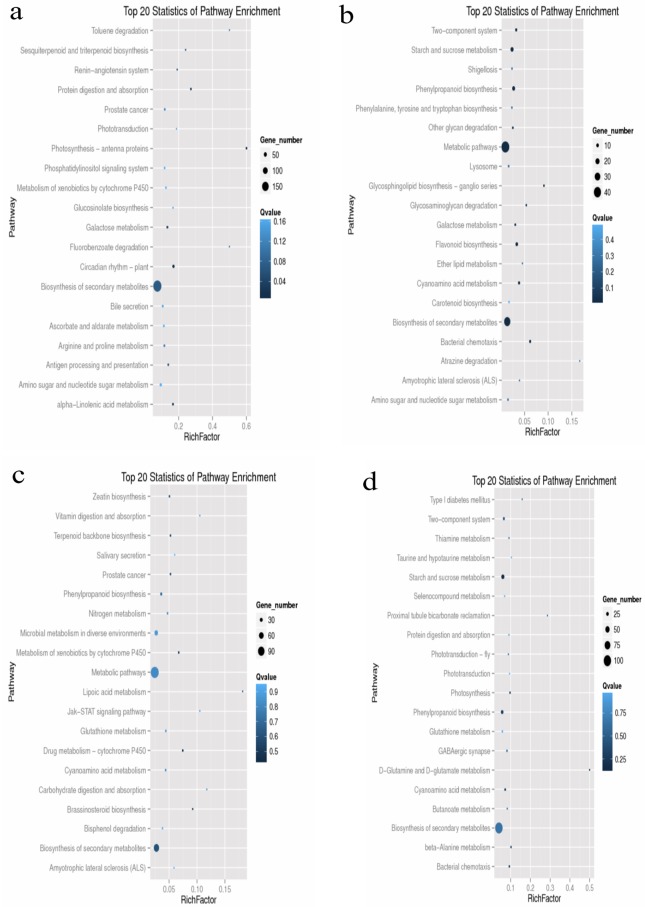
Scatter plot of KEGG pathway enrichment statistics of the comparison schemes. Top 20 statistics of pathway enrichment for Comparison A (a), Comparison B (b), Comparison C (c), Comparison D (d), respectively.

The DEGs detected in Comparisons A, B, C, and D were analyzed using the KEGG database to identify associated pathways. A KEGG enrichment analysis of DEGs from Comparisons A, B, C, and D involved 1,062, 103, 364, and 581 DEGs, accounting for 61.82%, 63.98%, 61.69%, and 64.48% of the total DEGs, respectively. The enriched pathways for the top 20 DEGs from Comparisons A, B, C, and D are provided in Fig 5. The top 20 DEGs were associated mainly with metabolic pathways (ko01100) and the biosynthesis of secondary metabolites (ko01110), as well as plant hormone signal transduction (ko04075), photosynthesis (ko00195), isoflavonoid biosynthesis (ko00943), and nitrogen metabolism (ko00910).

### Differentially expressed genes encoding transcription factors

The important roles of TFs in plant defenses against abiotic stresses in various species have been reported [37, 38]. In this study, we detected 202, 315, 273, and 364 DEGs encoding 237, 371, 323, and 446 TFs in the PT4S *vs*. PT2S, PT2S *vs*. PT2, PT4 *vs*. PT2, and PT4S *vs*. PT4 comparisons, respectively (S5 Table). These TFs were classified into 36, 42, 44, and 44 TF families in the four comparisons, including the AP2-EREBP, bHLH, GRAS, MYB, NAC, and WRKY TF families (Table 3). The PT4S *vs*. PT4 and PT2S *vs*. PT2 comparisons had the highest numbers of TFs and TF families. Interestingly, S1Fa-like and TIG, which may be salt-sensitive TFs, were detected only in the PT4S *vs*. PT4 and PT2S *vs*. PT2 comparisons. Additionally, GRF and BSD, which may be salt-tolerant TFs in the autotetraploid *P*. *tomentosa*, were identified only in the PT4S *vs*. PT2S and PT4 *vs*. PT2 comparisons.

**Table 3:** The number of DEGs in transcription factor families in response to salt stress treatments.

TF family	Number of genes											
	PT4S vs.PT2S			PT2S vs.PT2			PT4 vs.PT2			PT4S vs.PT4		
	Number	Up	Down	Number	Up	Down	Number	Up	Down	Number	Up	Down
ABI3VP1	2	0	2	3	2	1	3	0	3	6	3	3
Alfin-like	0	0	0	2	1	1	0	0	0	0	0	0
AP2-EREBP	24	7	17	33	7	26	32	9	23	38	14	24
ARF	7	3	4	5	5	0	12	8	4	5	4	1
ARR-B	3	1	2	4	2	2	2	0	2	3	3	0
BES1	0	0	0	3	3	0	1	0	1	2	2	0
bHLH	15	11	4	34	21	13	19	14	5	27	16	11
BSD	1	0	1	0	0	0	2	1	1	0	0	0
bZIP	0	0	0	6	4	2	3	1	2	6	4	2
C2C2-CO-like	4	3	1	7	6	1	7	3	4	8	7	1
C2C2-Dof	6	5	1	6	4	2	2	1	1	9	8	1
C2C2-GATA	1	0	1	5	4	1	5	4	1	4	1	3
C2C2-YABBY	0	0	0	0	0	0	0	0	0	2	1	1
C2H2	8	5	3	11	11	0	10	3	7	15	14	1
C3H	6	4	2	9	6	3	11	4	7	14	11	3
CAMTA	0	0	0	6	5	1	1	1	0	3	0	3
CPP	1	1	0	2	2	0	1	1	0	1	1	0
DBP	0	0	0	0	0	0	0	0	0	1	0	1
E2F-DP	0	0	0	1	1	0	0	0	0	0	0	0
EIL	0	0	0	2	0	2	3	0	3	0	0	0
FAR1	1	1	0		0	0	1	0	1	5	5	0
FHA	1	0	1	4	3	1	3	2	1	2	1	1
G2-like	7	5	2	12	7	5	7	2	5	15	11	4
GeBP	0	0	0	0	0	0	0	0	0	2	2	0
GRAS	13	8	5	15	8	7	11	4	7	23	19	4
GRF	2	2	0	0	0	0	1	1	0	0	0	0
HB	0	0	0	2	2	0	1	0	1	4	4	0
HSF	5	4	1	14	2	12	8	1	7	12	7	5
LIM	1	1	0	3	1	2	3	3	0	3	1	2
LOB	4	0	4	5	3	2	5	0	5	4	4	0
MADS	1	0	1	3	3	0	2	1	1	2	2	0
mTERF	6	3	3	5	5	0	8	8	0	1	0	1
MYB	33	18	15	52	29	23	48	32	16	77	40	37
MYB-related	26	12	14	35	18	17	40	29	11	57	25	32
NAC	22	11	11	18	12	6	19	4	15	19	17	2
PBF-2-like	0	0	0	0	0	0	1	1	0	0	0	0
PLATZ	1	0	1	2	1	1	2	0	2	2	2	0
PWP-PK	4	3	1	3	1	2	2	0	2	5	5	0
S1Fa-like	0	0	0	1	1	0	0	0	0	1	1	0
SBP	2	1	1	3	2	1	1	1	0	3	2	1
sigma70-like	1	1	0	6	2	4	4	3	1	3	0	3
SRS	0	0	0	0	0	0	1	1	0	0	0	0
TA2	2	1	1	4	1	3	1	0	1	3	1	2
TCP	2	0	2	2	1	1	3	0	3	4	3	1
Tify	1	0	1	5	0	5	3	2	1	5	1	4
TIG	0	0	0	4	3	1	0	0	0	3	3	0
Trihelix	3	3	0	8	7	1	7	3	4	12	11	1
TUB	0	0	0	2	1	1	3	1	2	3	3	0
VOZ	2	1	1	0	0	0	1	0	1	2	2	0
WRKY	17	7	10	22	8	14	22	3	19	28	16	12
zf-HD	2	2	0	2	2	0	1	1	0	2	2	0
Total	237	124	113	371	207	164	323	153	170	446	279	167

We detected 159, 8, 29, and 51 DEGs encoding 193, 9, 34, and 57 TFs from 37, 6, 18, and 21 families in Comparisons A, B, C, and D, respectively (S6 Table). The four common TF super-families among these comparisons were AP2/EREBP, bHLH, MYB, and NAC. Of these, the bHLH, MYB, and NAC TFs were mostly up-regulated in response to increased salt stress and ploidy level, whereas *AP2/EREBP* was mostly down-regulated.

### Differentially expressed genes involved in alternative splicing

**Fig 6:**
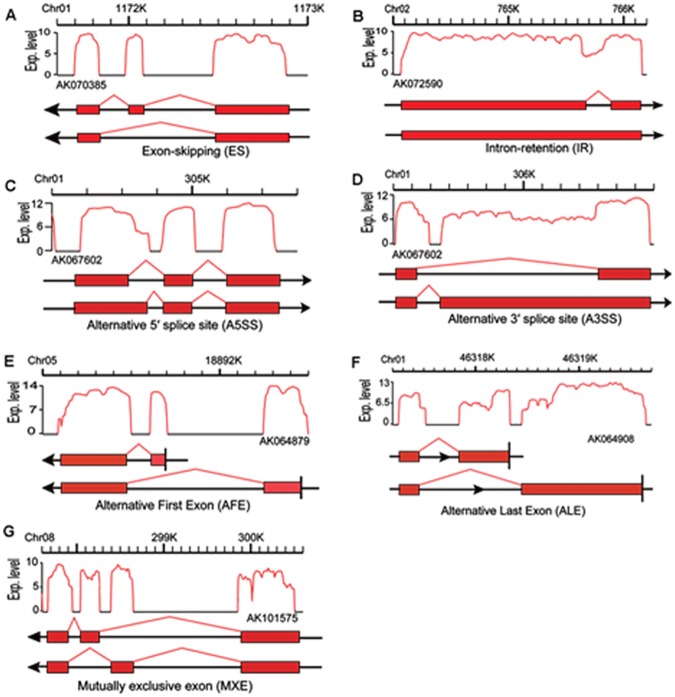
Alternative splicing events “Exp. Level” of Y-axis equals to log2 (Reads number).

**Fig 7:**
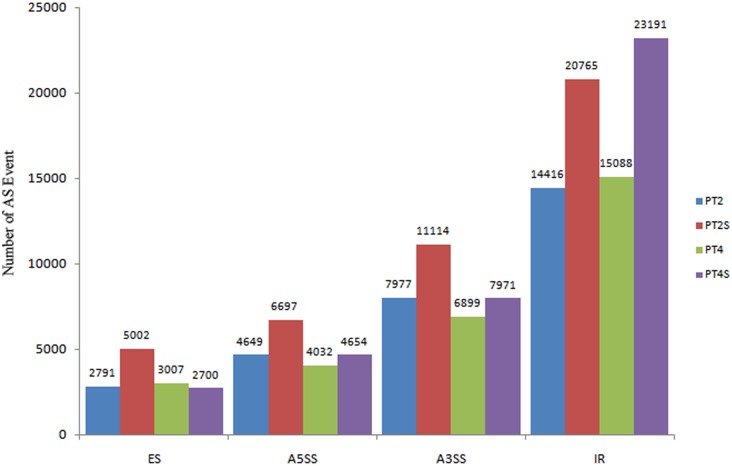
Classification of the four main AS events in this study.

AS affects many plant physiological processes, including responses to abiotic and biotic stresses [39]. There are seven main types of AS: Exon skipping (ES), Intron retention (IR), Alternative 5′ splice site (A5SS), and Alternative 3′ splice site (A3SS), Alternative first exon (AFE), Alternative last exon (ALE), and Mutually exclusive exon (MEX) (Fig 6). The AS of eukaryotic genes mainly involves the first four types. The last three types were excluded in this study because of the potential for considerable false-positive results. Among the four included AS categories, we observed that IR was the most common (73,460), followed by A3SS (33,961), A5SS (20032), and ES (13500) (Fig 7). That IR was the primary AS category was consistent with the findings of previous studies [40, 41]. Of the four main AS types, A5SS, A3SS, and IR were significantly enhanced in response to saline conditions. Furthermore, IR was especially enhanced in the PT4S *vs*. PT2S and PT4 *vs*. PT2 comparisons. These results implied that IR is influenced by the ploidy level.

### Differentially expressed genes involved in plant hormone signal transduction

Plant hormone signal transduction is crucial for plant responses to adverse environmental conditions. We detected 157 DEGs related to plant hormone signal transduction. In Comparison A, 90 DEGs were related to signal transductions involving several hormones, including ABA, GA, IAA, CK, ET, JA, SA, and BR, and in Comparisons B, C, and D 7, 20, and 40 DEGs were related to plant hormone signal transduction, respectively (S7 Table).

### Differentially expressed genes involved in photosynthesis

Exposure to high-salt conditions impairs osmotic homeostasis as well as the cellular ionic and redox balance, and also inhibits photosynthetic activities. In this study, the “photosynthesis, light reaction”, “photosynthetic membrane”, “chloroplast”, and “chloroplast part” GO terms were enriched in Comparisons A, B, C, and D. The GO analyses indicated that 228 DEGs were associated with “photosynthesis”. The KEGG pathway analyses revealed that photosystem II P680 reaction center D1 protein (psbA), photosystem II oxygen-evolving enhancer protein 3 (psbQ), photosystem I P700 chlorophyll *a* apoprotein A1 (psaA), photosystem I subunit PsaN (psaN), ferredoxin (petF), and ferredoxin-NADP^+^reductase (petH) were significantly enriched in the four comparisons (S8 Table).

### Validation of differentially expressed genes by quantitative real-time polymerase chain analysis

**Fig 8:**
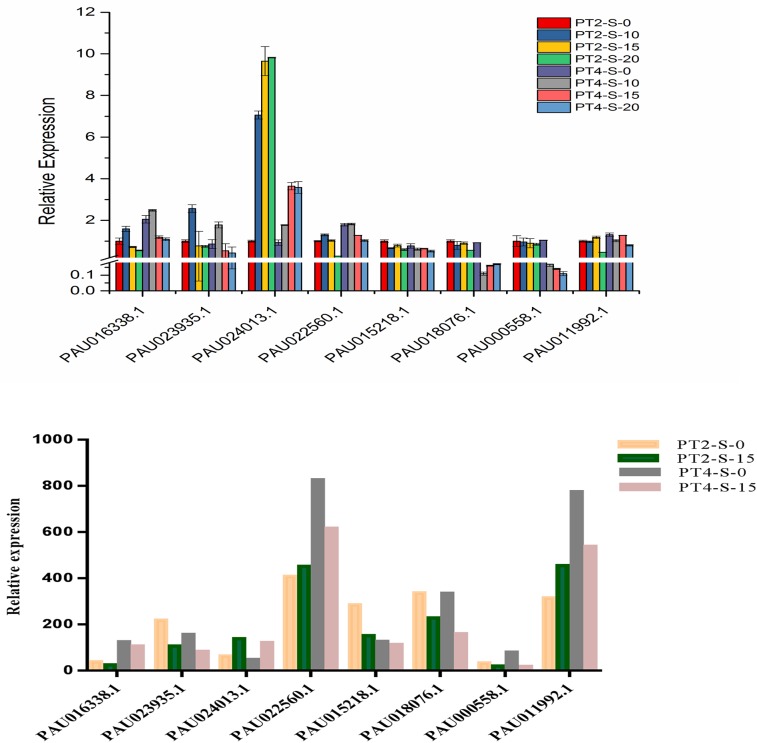
Expression pattern confirmation of selected genes by qRT-PCR. (A) Quantitative Real-Time PCR (qRT-PCR) analysis of 8 selected differentially expressed genes. 18S rRNA was used as the internal reference gene. For each group, the PT2 (S-0) expressed level was considered as 1.00, and other samples were normalized accordingly. Standard error of the mean for three technical replicates is represented by the error bars. S-0, S-10, S-15, and S-20 (0, 10, 15, and 20 days), 0.4% NaCl salt-treated for PT2 and PT4, respectively. (B) Changes in the relative expression levels of 8 selected genes as determined by RNA-Seq.

To further validate the reliability and reproducibility of the gene expression results from the RNA-Seq data, eight DEGs from the four libraries were selected for qRT-PCR analysis. The qRT-PCR results for the eight DEGs reflected the same trends (up- and down-regulation trends) as those acquired from the RNA-Seq data using the FPKM method (Fig 8A). The disaccord between the transcriptome and qRT-PCR results is likely because transcriptome data better reflect small changes in gene expression and better detection of low-abundant than qRT-PCR, as reported previously [42]. Overall, the qRT-PCR results support the reliability of the relative values provided by the RNA-Seq analysis.

The qRT-PCR results are related to the DEG data. The expression trends of all 8 genes from RNA-Seq and qRT-PCR analyses were largely consistent, demonstrating the correlation analysis of DEGs under salt stress based on RNA-Seq and qRT-PCR data (Fig 8B).

## Discussion

Salinity is a major environmental stressor that constrains tree growth in arid and semi-arid regions [43]. High-salt conditions can induce morphological and physiological changes in *P*. *tomentosa* leaves [20]. Currently, only limited information is available regarding the mechanism underlying the salt tolerance of *P*. *tomentosa*. In this study, the second pairs of leaves from the apex shoot of PT4S, PT4, PT2S, and PT2 seedlings were analyzed by RNA-Seq to elucidate the complex mechanisms that regulate the responses of diploid and autotetraploid *P*. *tomentosa* to salt-stress conditions. The transcriptome data yielded 175 million clean reads, many of which were differentially expressed. Overall, the control plants had fewer total mapped reads than the salt-treated plants (i.e., PT2 < PT2S and PT4 < PT4S) (Table 2). Additionally, some overlapping DEGs were detected. A comparison of the control and salt-treated *P*. *tomentosa* plants of different ploidy levels decreased the number of DEGs. This increased the likelihood that the identified genes were related to plant responses and adaptations to saline conditions. “Plant hormone signal transduction” (ko07075) and “Photosynthesis” (ko00195) were considerably enriched metabolic pathways. The enriched TFs may be useful for clarifying the molecular mechanism underlying the salt tolerance of the diploid and autotetraploid *P*. *tomentosa* lines.

### Transcription factors involved in salt stress responses

Transcription factors are important for the acclimation of plants to extreme environmental stresses, including high-salt conditions. In this study, members of the bHLH, MYB, NAC, GRAS, WRKY, and AP2/EREBP families were the most abundant stress- or ploidy-related TFs. Some calcium-dependent TFs are regulated by the direct binding of a Ca^2+^ ion or the Ca^2+^/calmodulin (CaM) complex or by post-translation modifications mediated by Ca^2+^ or the Ca^2+^/CaM complex [44]. AtNIG1 (*Arabidopsis thaliana* NaCl-inducible gene 1), which is a basic helix–loop–helix (bHLH) TF, was the first identified salt stress-responsive Ca^2+^-binding TF involved in salt stress signaling in plants according to a suppression subtractive hybridization analysis [45]. In the current study, we identified 62 up-regulated and 33 down-regulated *bHLH* genes that were induced by salt stress, which is consistent with the results of a previous study in *A*.*thaliana* [46]. The MYB super family, which includes MYB and MYB-related TFs, was the largest TF family in our study. MYB TFs have regulatory roles in plant developmental processes and defense responses [47]. A two-repeat MYB (i.e., MYB2), which contains a Ca^2+^-dependent CaM-binding domain, regulates the expression of dehydration- and salt-responsive genes in *A*. *thaliana* [48]. Additionally, the over-expression of *OsMYB3R*-2 in *A*. *thaliana* [49] and *OsMYB48-1* in rice [50] reportedly enhanced tolerance to high-salt conditions. We detected 203 up-regulated and 165 down-regulated *MYB* genes in our transcriptome data. The NAC TFs, which form one of the largest plant-specific TF families, are essential for responses to various abiotic stresses. A NAC domain-containing TF was identified as a CaM-binding protein. Analyses of transcriptional regulation mediated by a CaM-binding NAC protein helped characterize the function of the Ca^2+^/CaM complex [51]. The over-expression of *TaNAC2* in *A*. *thaliana* plants led to increased tolerance to salt, drought, and freezing stresses [52]. We observed that *NAC* expression levels were mostly up-regulated in salt-stressed *P*. *tomentosa* plants. This is consistent with the results of a previous study in cotton [53]. These DEGs may be relevant for future studies of *P*. *tomentosa* salt tolerance.

### Plant hormone signal transduction pathways involved in salt stress responses

Phytohormones influence plant responses to abiotic stresses. For example, to optimize the chance of survival, phytohormone production may be altered to decrease plant growth, enabling the plant to divert additional resources to maintain responses to environmental stresses [54]. Indeed, the perception of a stress signal causes the phytohormone-related signal transduction cascades in plants to decrease to baseline levels [55, 56].

ABA is a lipophilic plant hormone with key roles in signaling and adaptation to abiotic stresses, such as drought and high salinity [57]. The ABA signal transduction pathway involves the activities of PP2C (Proteinphosphatases 2C), PYR/PYL (abscisic acid receptor PYR/PYL family), SnRK2 (serine/threonine-protein kinase SRK2), and ABF (ABA responsive element binding factor). This pathway is initiated when ABA binds to the pyrabactin resistance-like/regulatory component of ABA receptors (RCAR). Which results in the inactivation of protein phosphatase PP2C and activation of SnRK2-type kinases, ultimately inducing stomatal closure [58]. As a positive regulator of ABA signaling, SnRK2 is crucial for abiotic stress responses in plants [59]. In this study, *SnRK2* expression was up-regulated in the PT4 *vs*. PT2 and PT4S *vs*. PT4 comparisons (S7 Table), similar to the results of a previous study of maize [60]. There is genetic evidence that A-type PP2Cs are negative regulators of ABA signaling in *A*.*thaliana* [61]. Under saline conditions, *ABF2* expression is reportedly up-regulated in grape leaves and roots in response to increasing salt concentrations [62]. Thus, the expression of salt-responsive genes may be related to the down-regulation of PP2C and up-regulation of ABF associated with ABA signal transduction. In this study, *PP2C* expression (PAU005151.1, PAU008363.1, and PAU024054.1) was down-regulated in the PT4 *vs*. PT2 and PT4S *vs*. PT2S comparisons. In contrast, *ABF* (PAU013700.1, PAU023164.1, and PAU013700.1) expression levels increased in salt-stressed PT2S and PT4S plants.

Cytokinins (CKs) are a class of phytohormones involved in various physiological events, including responses to environmental stresses. Recently, CKs were revealed to regulate plant adaptations to salt stress during growth and development [63], and increased CK levels in seeds were observed to increase plant tolerance to salt stress [64]. The CRE1 histidine kinase (the cytokinin response 1), which contains an extracellular domain called CHASE, is a CK receptor. The CK signaling pathway involves several steps [65]. First, CRE1 is activated when CK binds to the CHASE domain and isauto-phosphorylated. Then, histidine-containing phosphotransfer proteins (AHPs) are phosphorylated by the activated CRE1,and migrate from the cytoplasm to the nucleus, where they transfer the phosphate group to (the response regulator proteins) ARRs. The expression of *CRE1* (one of the receptor histidine kinases) is induced by various stresses, suggesting that the histidine kinase CK receptors are crucial for CK and stress responses in *A*. *thaliana* [66]. In this study, *CRE1* expression (PAU010104.1 and PAU010665.1) was up-regulated in the PT2S *vs*. PT2 and PT4S *vs*. PT4 comparisons, which is in agreement with the aforementioned results. Furthermore, *CRE1* expression (PAU021126.1) was also up-regulated in the PT4 *vs*. PT2 and PT4S *vs*. PT2S comparisons, which may be related to differences in ploidy levels. Nishiyama et al. [67] reported that among genes related to CK signaling, the expression of a type-B *ARR* gene was down-regulated, whereas *AHP4* was up-regulated in response to salt treatments. This implies that CK signaling might be inhibited by salt stress. We observed that type-B *ARR* expression (PAU012990.1) levels were down-regulated in the PT2S *vs*. PT2 and PT4S *vs*. PT4 comparisons, which is consistent with the aforementioned findings. Type-B *ARR* expression (PAU003823.1, PAU001788.1, PAU022082.1, and PAU022609.1) was down-regulated in the PT4 *vs*. PT2 and PT4S *vs*. PT2S comparisons, which may be related to the variability in ploidy levels. The expression of *AHP* (PAU029211.1) was up-regulated in the PT2S *vs*. PT2 and PT4S *vs*. PT4 comparisons, which is consistent with the previously described results.

In this study, we identified 157 DEGs related to plant hormone signal transduction. The results of this study along with previously reported findings revealed that autotetraploid *P*. *tomentosa* was more salt-tolerant than diploid *P*. *tomentosa*. We observed that all the analyzed plant hormones were directly or indirectly involved in regulating plant responses to salt stress. The activities of plant genes and hormones are strongly linked, as indicated by the fact that some plant genes, which are essential for activating plant hormones and other genes, are activated by phytohormones.

### Photosynthetic activities involved in salt stress responses

Salt stress decreases plant growth and productivity by disrupting physiological processes [68], especially photosynthetic activities [69]. In this study, several DEGs were involved in photosynthesis, including genes encoding the photosystem II P680 reaction center D1 protein (psbA), photosystem II oxygen-evolving enhancer protein 3 (psbQ), photosystem I P700 chlorophyll *a* apoprotein A1 (psaA), ferredoxin (petF), and ferredoxin-NADP^+^ reductase (petH). Thylakoid membrane proteins were influenced by salt stress in *Synechococcus* sp.PCC 7942 [70]. The photosystem II D1 protein in thylakoid membranes is encoded by a gene in the plastid genome (cpDNA). This protein transforms radiant energy via the oxidation of water and reduction of plastoquinone. In *Synechocystis* sp., salt stress suppresses the repair of photosystem II by inhibiting the activities of the transcriptional and translational machinery [71]. Additionally, the D1 protein is sensitive to environmental stresses [72]. We observed that the expression of the gene (PAU015578.1), which encoded a D1 protein, was down-regulated in the PT4S *vs*. PT4 and PT2S *vs*. PT2 comparisons (S8 Table). This is consistent with the previous observation that D1 levels decreased because of salt stress-induced photoinhibition in *Brassica juncea* [73]. The psbQ peripheral protein is an important part of the photosystem II complex. It is also the most diverse extrinsic photosystem II protein in higher plants, and can maintain the integrity of photosystem II under high-salt conditions [74]. The *psaQ* expression level was up-regulated in the PT4 *vs*. PT2 and PT4S *vs*. PT2S comparisons, which may be closely related to the differences in ploidy. The *psaA* gene encodes photosystem I P700 chlorophyll *a* apoprotein A1, which is one of two large core subunits of photosystem I. It carries a set of cofactors required for a functional electron transport chain through photosystem I. In this study, the abundance of the *psaA* transcript (PAU029948.1) was up-regulated in the PT2S *vs*. PT2 comparison. This is in agreement with the results of a previous study of *Salicorniabigelovii* Torr [75]. Electrons derived from water by the oxygen-evolving complex of photosystem II are transferred to NADP^+^, leading to the production of NADPH. The electrons are passed along the photosynthetic electron transport chain *via* the plastoquinone, cytochrome b_6_f complex, plastocyanin, photosystem I, ferredoxin, and ferredoxin-NADP^+^oxidoreductase [76].

## Conclusions

Here we report for the first time, comparisons of the effects of salt stress on diploid and autotetraploid *P*. *tomentosa* plants using Illumina sequencing technology based on the *P*. *fortunei* genome as a reference. Overall, 85.7 and 125 million clean reads were mapped to *P*. *fortunei* gene and genome sequences, respectively. We identified 15,873 million DEGs among the control and salt-stressed *P*. *tomentosa* plants. Some DEGs were functionally associated with plant hormone signal transductions, photosynthesis, and other metabolic pathways. Notably, some TF genes, including *NAC*, *MYB*, *bHLH*, *GRAS*, *WRKY*, and *AP2/EREBP*, were responsive to salt stress and/or ploidy levels. We also identified several DEGs related to AS which were affected by salt stress or ploidy levels. Our results may be useful for future efforts to elucidate the molecular mechanisms underlying salt tolerance as well as the gene regulatory networks of *P*. *tomentosa* plants.

## Supporting information

S1 Fig Gene ontology analysis of DEGs in comparisons schemes: Comparison A (a), comparison B (b), comparison C (c), and comparison D (d). (TIF)

S1 Table Correlation value between each two samples. (DOCX)

S2 Table The DEGs in the four comparisons: PT4S vs. PT2S, PT2S vs. PT2, PT4S vs. PT4, and PT4 vs. PT2. (XLSX)

S3 Table The DEGs in the comparisons schemes: Comparison A, comparison B, comparison C, and comparison D. (XLSX)

S4 Table The gene 2GO of the comparisons schemes: Comparison A, comparison B, comparison C, and comparison D. (XLSX)

S5 Table The transcription factors of DEGs in the four comparisons: PT4S vs. PT2S, PT2S vs. PT2, PT4S vs. PT4, and PT4 vs. PT2. (XLSX)

S6 Table The transcription factors of DEGs in the comparisons schemes: Comparison A, comparison B, comparison C, and comparison D. (XLSX)

S7 Table The plant hormone signal transduction of DEGs in the comparisons schemes: Comparison A, comparison B, comparison C, and comparison D. (XLSX)

S8 Table The photosynthesis of DEGs in the comparisons schemes: Comparison A, comparison B, comparison C, and comparison D. (XLS)
