# Supplementary figures and images for: Genome-wide expression analysis of salt-stressed diploid and autotetraploid Paulownia tomentosa
Source: PLoS One. 2017 Oct 19;12(10):e0185455. doi: 10.1371/journal.pone.0185455 (PMC5648118; doi:10.1371/journal.pone.0185455)

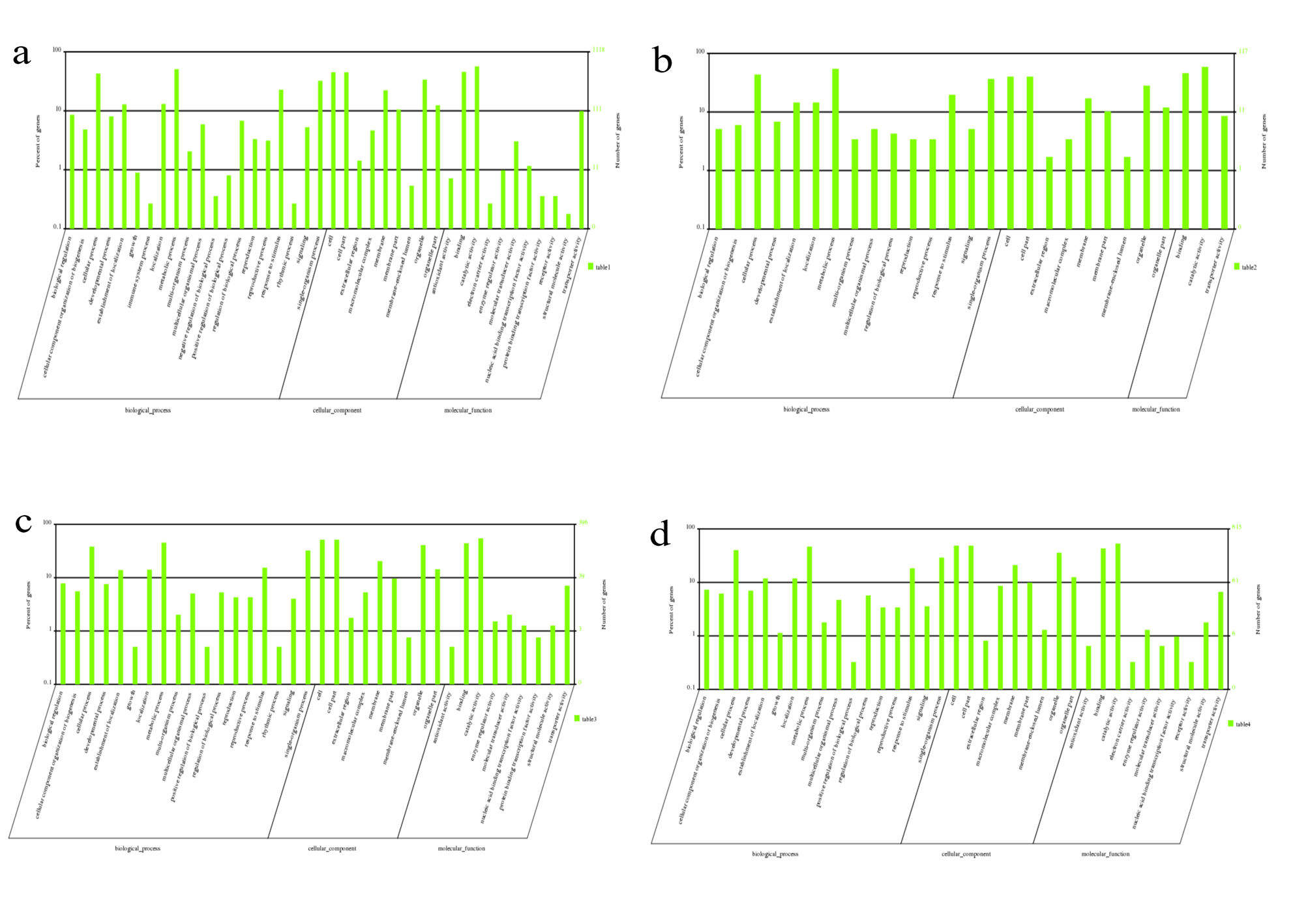

Supplement: S1 Fig — (TIF) [file pone.0185455.s001.tif]
