# Supplementary material for: Genome-wide expression analysis of salt-stressed diploid and autotetraploid Paulownia tomentosa
Source: PLoS One. 2017 Oct 19;12(10):e0185455. doi: 10.1371/journal.pone.0185455 (PMC5648118; doi:10.1371/journal.pone.0185455)
Supplement: S1 Table — (DOCX) [file pone.0185455.s002.docx]

| Sample | PT2S | PT2 | PT4 | PT4S |
| --- | --- | --- | --- | --- |
| PT2S | 1 | 0.710672 | 0.758158 | 0.837682 |
| PT2 | 0.710672 | 1 | 0.738544 | 0.68499 |
| PT4 | 0.758158 | 0.738544 | 1 | 0.864472 |
| PT4S | 0.837682 | 0.68499 | 0.864472 | 1 |

Table S1. Correlation value between each two samples.
